# Serum Selenium-Binding Protein 1 (SELENBP1) in Burn Injury: A Potential Biomarker of Disease Severity and Clinical Course

**DOI:** 10.3390/antiox12111927

**Published:** 2023-10-29

**Authors:** Tabael L. Turan, Holger J. Klein, Julian Hackler, Livia Hoerner, Eddy Rijntjes, Theresia Reding Graf, Jan A. Plock, Lutz Schomburg

**Affiliations:** 1 Institute for Experimental Endocrinology, Max Rubner Center for Cardiovascular Metabolic Renal Research, Charité—Universitätsmedizin Berlin, Corporate Member of Freie Universität Berlin, Humboldt-Universität zu Berlin, and Berlin Institute of Health, 10115 Berlin, Germany; tabael-lee.turan@charite.de (T.L.T.); livia.hoerner@charite.de (L.H.); eddy.rijntjes@charite.de (E.R.); 2 Department of Plastic Surgery and Hand Surgery, University Hospital Zurich, 8091 Zurich, Switzerland; holger.klein@ksa.ch (H.J.K.); jan.plock@ksa.ch (J.A.P.); 3 Department of Plastic Surgery and Hand Surgery, Cantonal Hospital Aarau, 5001 Aarau, Switzerland; 4 Department of Visceral Surgery and Transplantation, University Hospital Zurich, 8091 Zurich, Switzerland

**Keywords:** selenium, trace element, critical disease, pneumonia, prognosis, critical care

## Abstract

Oxidative stress, systemic inflammation, and metabolic derangements are hallmarks of burn pathophysiology. Severely burned patients are highly susceptible to infectious complications. Selenium-binding protein 1 (SELENBP1) modulates intracellular redox homeostasis, and elevated serum concentrations have been associated with adverse clinical outcomes in trauma patients. We hypothesized that serum SELENBP1 at hospital admission and during hospitalization may constitute a meaningful biomarker of disease severity and the clinical course in burn injury, with pulmonary infection as primary endpoint. To this end, we conducted a prospective cohort study that included 90 adult patients admitted to the Burn Center of the University Hospital Zurich, Switzerland. Patients were treated according to the local standard of care, with high-dose selenium supplementation during the first week. Serum SELENBP1 was determined at nine time-points up to six months postburn and the data were correlated to clinical parameters. SELENBP1 was initially elevated and rapidly declined within the first day. Baseline SELENBP1 levels correlated positively with the Abbreviated Burn Severity Index (ABSI) (R = 0.408; *p* < 0.0001). In multiple logistic regression, a higher ABSI was significantly associated with increased pulmonary infection risk (OR, 14.4; 95% CI, 3.2–88.8; *p* = 0.001). Similarly, baseline SELENBP1 levels constituted a novel but less accurate predictor of pulmonary infection risk (OR, 2.5; 95% CI, 0.7–8.9; *p* = 0.164). Further studies are needed to explore the additional value of serum SELENBP1 when stratifying patients with respect to the clinical course following major burns and, potentially, for monitoring therapeutic measures aimed at reducing tissue damage and oxidative stress.

## 1. Introduction

Selenium-binding protein 1 (SELENBP1) is among the diverse group of selenium (Se)-containing proteins. Unlike the proteins containing selenocysteine (Sec) or selenomethionine, SELENBP1 directly binds Se [1]. While it was first discovered in 1989 [2], the role of SELENBP1 under physiological and pathological conditions still remains poorly understood [3]. However, it may participate in several physiological processes, such as cell differentiation [4,5], proteasomal protein degradation [6], intra-Golgi transport [7], and redox modulation by its oxidoreductase activity [8], with copper (Cu) as a required cofactor [9]. SELENBP1 does not appear to be induced by dietary Se exposure, as shown in rodents [10] and the nematode *C. elegans* [9]. It is ubiquitously expressed and subcellularly localized in both the nucleus and the cytoplasm [11]. Intracellularly, SELENBP1 affects redox homeostasis by reciprocal interference with the antioxidative activity of the selenoenzyme glutathione peroxidase 1 (GPx-1) [12,13]. Downregulation of SELENBP1 expression has been associated with carcinogenesis and the poor prognosis of various human malignancies [3]. Furthermore, SELENBP1 has been shown to be upregulated in the prefrontal cortex of patients with schizophrenia [14], even causing and augmenting negative symptoms [15]. Elevated serum levels of SELENBP1 have been detected and correlated with adverse clinical outcomes in patients with traumatic spinal cord injury [16], acute coronary syndrome [17], and following cardiac surgery [18].

Severe burns entail immediate inflammatory and metabolic alterations that persist for years after the injury [19]. Long-term inflammation is largely maintained by burn-induced systemic oxidative stress [20,21]. Acute Se depletion is frequent after major burns [22], and trace element supplementation is associated with an improved clinical outcome [23]. Due to metabolic derangements, skin barrier disruption, exposure to invasive procedures, and prolonged hospitalization, burn patients are particularly prone to infectious complications [24,25]. Sepsis and preceding infections, pulmonary infections in particular, are the leading cause of morbidity and mortality among burn patients [26,27,28]. With that in mind, the importance of expanding strategies to predict and prevent infections in the early postburn period comes into focus.

In the present trial, we report on the dynamics of serum SELENBP1 expression in burn patients and examine whether it relates to the severity of the injury. We also address our hypothesis that higher baseline levels of serum SELENBP1 may be predictive of an adverse clinical outcome in these patients, accentuating pulmonary infection as a main source of mortality.

## 2. Materials and Methods

### 2.1. Study Design

A total of 90 adult patients (≥18 years old) admitted to the Burn Center of the University Hospital Zurich, Switzerland, were prospectively recruited between May 2015 and October 2018. Exclusion criteria were current infection at admission, immunosuppressive medication, and burn injuries older than 6 h.

Burn severity was assessed using the Abbreviated Burn Severity Index (ABSI), a predictive score for burn mortality consisting of five variables: sex, age, presence of inhalation injury, presence of full-thickness burn, and percentage of the total body surface area (TBSA) burned. The patients can thereby be stratified into six groups with varying degrees of injury [29]. Inhalation injury refers to an acute respiratory tract involvement caused by exposure to thermal or chemical noxae. Full-thickness burns affect all layers of the skin, including the epidermis, dermis, and subcutis. Affected TBSA was determined using Lund–Browder charts. Patients were treated in accordance with the standard of care, which included surgical interventions, intravenous fluid resuscitation, early enteral nutrition, a high-protein diet, and regular indirect calorimetry to estimate caloric requirements. In addition, all patients received 1000 μg of Se per day from admission to day 7 inclusively, which was administered by the continuous infusion of sodium selenite. Continuation of intravenous Se supplementation with 500 μg per day was conducted in unstable patients until clinical stability was reached. Serum Cu and zinc (Zn) levels were determined once a week and supplemented based on personal need until discharge.

The primary clinical outcome measure was the development of pulmonary infection during hospitalization. A pneumonia diagnosis required a new pulmonary infiltrate to be detected on the chest radiograph, accompanied by relevant clinical manifestations, e.g., fever, cough, purulent expectoration, or dyspnea. Secondary clinical outcomes were in-hospital mortality, hospital length of stay (LOS), intensive care unit (ICU) LOS, and sepsis occurrence, based on Sepsis-3 [30]. We additionally recorded demographic characteristics, comorbidities, and trauma-related data.

The trial was registered at ClinicalTrials.gov (NCT02537821) on 2 September 2015.

### 2.2. Blood Sampling and Measurements

Blood samples were drawn upon admission (D0) and at the following time-points postburn: days (D) 1, 2, and 3; weeks (W) 1 and 2; and months (M) 1, 3, and 6. A set of *n* = 598 serum samples was prepared and stored at –80 °C until analyzed. In a subset of patients, post-discharge follow-up sampling was also carried out. Within the first two weeks, less than 10% of the values were missing; 6 months postburn, blood sampling had been successfully performed in 37% of patients.

Measurements were conducted in the laboratories of the Institute for Experimental Endocrinology, Charité—Universitätsmedizin Berlin, by technicians and researchers blinded to all the clinical data of the study population. Serum SELENBP1 concentrations were determined by a luminometric immunoassay as described earlier [17]. Moreover, the following serum parameters were evaluated by standardized procedures: trace elements (Se, Cu, Zn) by total reflection X-ray fluorescence (TXRF) [31], selenoprotein P (SELENOP) by a validated immunoluminometric sandwich assay (selenOtest ELISA; selenOmed GmbH, Berlin, Germany) [32], and GPx-3 activity by monitoring NADPH consumption in a coupled enzymatic test [33]. Inflammatory markers, such as white blood cells (WBCs), C-reactive protein (CRP), and procalcitonin (PCT), were measured by laboratories of the University Hospital Zurich as part of routine clinical procedures.

### 2.3. Statistical Analyses

Statistical analyses were performed using GraphPad Prism (Version 10.0.0; GraphPad Software, Inc., San Diego, CA, USA). Normality of the data was tested by the D’Agostino–Pearson test, and normally and non-normally distributed continuous data were expressed as means ± standard deviations (SD) and medians with interquartile range (Q1–Q3), respectively. Categorical variables were reported as absolute numbers and percentages (%). As appropriate, two groups were compared using the Student’s *t*-test, the Mann–Whitney U test, or Fisher’s exact test. Correlations were assessed by Spearman’s rank correlation coefficient. SELENBP1 time courses were compared between groups using a repeated measures mixed-effects model. Post hoc analyses for differences between groups were carried out by the Šidák test. Receiver-Operator-Curve (ROC) analysis was conducted to determine the validity of parameters that potentially predicted the development of pulmonary infection in burn patients. Thereafter, multiple logistic regression was performed to estimate the odds of developing pulmonary infection by baseline SELENBP1 levels and the ABSI. Patients developing infections other than pneumonia were excluded from both ROC and regression analyses. Two-tailed *p*-values < 0.05 were considered statistically significant: * *p* < 0.05, ** *p* < 0.01, *** *p* < 0.001, and **** *p* < 0.0001.

## 3. Results

### 3.1. Patient Demographic and Clinical Characteristics

The demographic and clinical characteristics of the participants are reported in Table 1. A total of 90 patients were included in this study, out of which 37 developed a pulmonary infection at a median (IQR) of 4 (3–6) days after admission. Twenty-two patients developed other infectious complications, namely cutaneous infections (*n* = 9), catheter-related infections (*n* = 7), bloodstream infections (*n* = 4), or urinary tract infections (*n* = 2). The majority of patients were male (73/90, 81.1%). The mean (± SD) age was 45.7 (± 17.7) years. Female patients suffered more severe burns than male patients, as evidenced by a markedly higher ABSI (9.4 ± 3.0 vs. 6.9 ± 2.2; *p* = 0.001). Compared with patients without infections, those with an accompanying pulmonary infection displayed a higher TBSA and a larger fraction of full-thickness burns and inhalation injuries, resulting in a significantly higher median [IQR] ABSI (8.0 [7.0–10.0] vs. 6.0 [4.0–7.0]; *p* < 0.0001). Sepsis occurred in almost all pneumonia patients (36/37, 97.3%). The enrolled patients had a median LOS of 27 days, with an expected longer duration of hospitalization in burn patients with a pulmonary infection.

The median [IQR] serum concentration of SELENBP1 at baseline was found to be considerably higher in the group of patients developing pulmonary infections than in burn patients without infections (45.6 [21.2–70.5] vs. 12.5 [3.7–34.2] µg/L; *p* = 0.007). Out of 31 patients without infections, 5 baseline samples were below the detection limit of the assay. In contrast, none of the patients who developed pneumonia were initially SELENBP1-negative.

### 3.2. SELENBP1 Is Elevated following a Severe Burn

**Figure 1:**
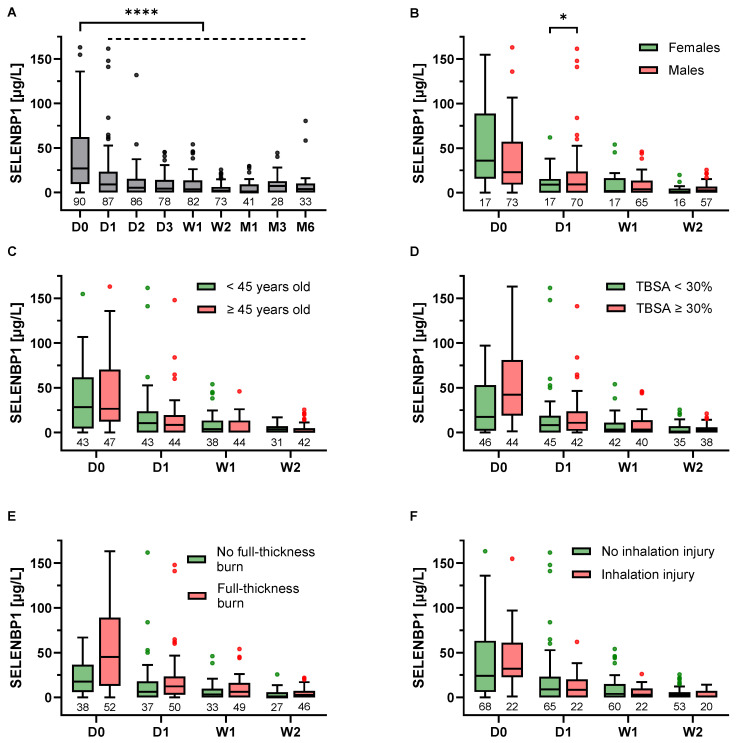
Time course of serum SELENBP1 in burn patients. (**A**) SELENBP1 levels were elevated upon admission (D0) but significantly dropped within the first day (D1) postburn and remained constant from then on. Patient stratification (**B**–**F**) revealed initially higher SELENBP1 levels in (**B**) females than in males, and in patients with (**D**) a total body surface area (TBSA) ≥ 30% vs. < 30% and (**E**) full-thickness burns vs. no full-thickness burns. Stratification by age (**C**) and the presence of inhalation injury (**F**) did not show considerable differences. SELENBP1 decreased more strongly in female patients compared with males, potentially explaining the significantly higher levels in males on day 1 (*p* = 0.048) (**B**). Results are presented as Tukey-style box plots. The numbers below the boxes indicate the number of patients in each group. The Y-axis-limit was set at 175 µg/L for optimal visualization. Statistical comparisons were conducted by a repeated measures mixed-effects model and the Šidák test for post hoc analyses. * *p* < 0.05, **** *p* < 0.0001.

On admission, serum SELENBP1 was considerably elevated in burn patients with a median (IQR) concentration of 26.9 (10.0–61.0) µg/L. Within the first posttraumatic day, serum levels declined significantly to a median (IQR) of 9.0 (0–22.5) µg/L. Thereafter, SELENBP1 remained at low levels throughout the observation period (Figure 1A). Stratified analysis by baseline characteristics revealed non-significantly higher initial SELENBP1 levels in females (Figure 1B) and burn patients with a TBSA ≥ 30% (Figure 1D) and full-thickness burns (Figure 1E). No such associations were found for stratification by age (Figure 1C) or the presence of inhalation injury (Figure 1F).

### 3.3. Baseline SELENBP1 Is Positively Associated with Burn Severity

**Figure 2:**
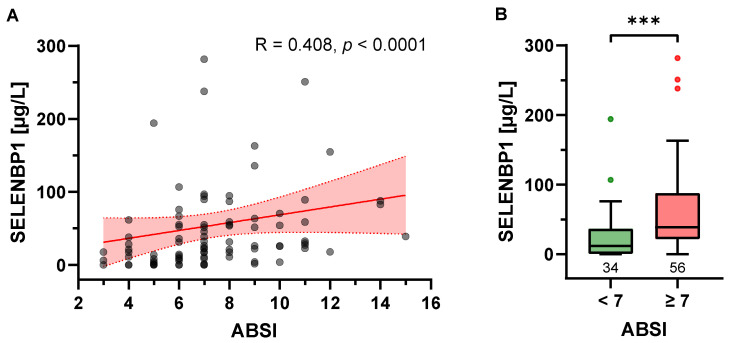
Correlation of baseline serum SELENBP1 concentration and burn severity. (**A**) Serum SELENBP1 concentration at baseline and the Abbreviated Burn Severity Index (ABSI) displayed a significant positive correlation, as visualized by a scatter plot and trend line (red) with 95% confidence intervals (red shadow); R: Spearman’s correlation coefficient, two-tailed. (**B**) When grouped according to their ABSI, burn patients with an ABSI ≥ 7 exhibited significantly higher SELENBP1 on admission than patients with an ABSI < 7 (*p* = 0.0002). Results are presented as Tukey-style box plots. The numbers below the boxes indicate the number of patients in each group. The Y-axis limit was set at 300 µg/L for optimal visualization; 2 data points exceeding 300 µg/L are missing in the figures ([7, 425.17], [7, 451.68]). *** *p* < 0.001.

A moderate positive correlation was found between serum SELENBP1 levels on admission and the ABSI (R = 0.408; *p* < 0.0001) (Figure 2A). The median (IQR) baseline SELENBP1 concentration was 11.7 (1.3–34.9) µg/L and 38.9 (22.0–87.2) µg/L in burn patients with an ABSI < 7 (*n* = 34) and ≥ 7 (*n* = 56), respectively (*p* = 0.0002) (Figure 2B). Interestingly, a groupwise Spearman’s correlation between the baseline SELENBP1 concentration and the ABSI by clinical characteristics revealed stronger associations in less severely affected patients: R = 0.339 (*p* = 0.021) vs. R = 0.198 (*p* = 0.198) in patients with TBSA < 30% vs. ≥ 30%; R = 0.444 (*p* = 0.005) vs. R = 0.138 (*p* = 0.329) in patients without vs. with full-thickness burns; and R = 0.390 (*p* = 0.001) vs. R = 0.311 (*p* = 0.159) in patients without vs. with inhalation injury, respectively (not shown). Moreover, baseline SELENBP1 levels and the ABSI correlated more strongly in patients aged < 45 years than in patients aged ≥ 45 years: R = 0.584 (*p* < 0.0001) vs. R = 0.157 (*p* = 0.292), respectively. No significant difference was observed between men and women.

### 3.4. Correlation Analysis of SELENBP1

An exploratory correlation analysis was conducted between SELENBP1 and selenium status biomarkers, inflammatory parameters, and trace elements (Table 2). SELENBP1 showed a weak inverse correlation with parameters of Se status, including significant correlation coefficients over the first two days after admission for Se and SELENOP. No significant association with Cu or Zn was found, regardless of the time postburn. In line with the ABSI results, positive correlations of SELENBP1 with inflammatory markers were observed, e.g., WBCs and PCT on admission. SELENBP1 and CRP showed a weak positive correlation on D2.

### 3.5. Baseline SELENBP1 and Burn Severity Are Associated with the Risk of Pulmonary Infection

**Figure 3:**
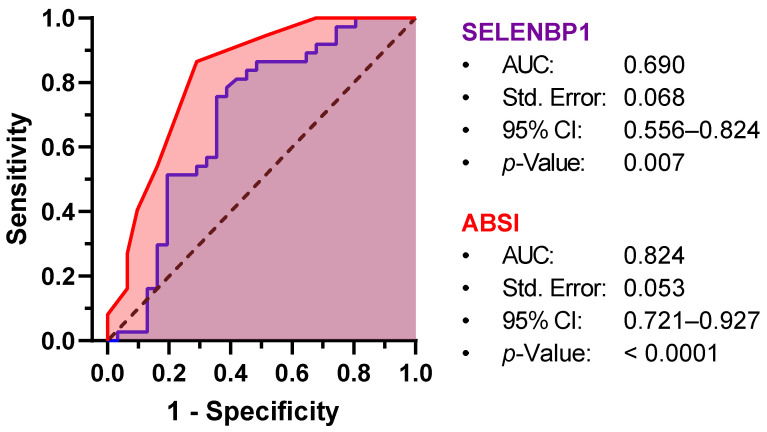
Receiver-Operator-Curve (ROC) analysis for the prediction of pulmonary infection by baseline SELENBP1 and ABSI. Both SELENBP1 (purple) and ABSI (red) are significantly associated with pulmonary infection risk, yielding areas under the ROC curve (AUC) of 0.690 and 0.824, respectively. Std. Error, standard error; CI, confidence interval.

To assess the potential value of baseline SELENBP1 concentration and the ABSI as predictors of pulmonary infection risk in burn patients, an ROC analysis was conducted, with an estimated area under the ROC curve (AUC) of 0.69 for SELENBP1 and 0.82 for the ABSI (Figure 3). Baseline levels of serum SELENBP1 > 21.2 µg/L predicted the risk of pulmonary infection with a sensitivity of 75.7% and a specificity of 64.5%. The ABSI has proven to be a more accurate predictor, with a sensitivity of 86.5% and a specificity of 71.0% at a cut-off of 6.5 (Table 3). Multiple logistic regression confirmed both baseline SELENBP1 and the ABSI as predictors of pulmonary infection in burn patients (Table 4). Burn patients with an ABSI of 7 or higher had a 14.4-fold greater risk of pulmonary infection than patients with an ABSI equal to or less than 6 (OR, 14.37; 95% CI, 3.15–88.78; *p* = 0.001). Initial SELENBP1 levels > 21.2 µg/L were associated with a 2.5-fold greater risk of pulmonary infection, although this did not reach statistical significance (OR, 2.47; 95% CI, 0.68–8.88; *p* = 0.164).

After adjusting for baseline SELENBP1 concentration and patient characteristics, changes in serum SELENBP1 concentrations within the first day after injury were not associated with an increased risk of pulmonary infection. Excluding female patients in multiple logistic regression did not yield significant changes regarding the predictive value of baseline SELENBP1 levels for pulmonary infection risk in burn patients.

## 4. Discussion

In this prospective cohort study, we observed an immediate systemic elevation of SELENBP1 after major burns. In line with previous studies reporting increased serum SELENBP1 levels following tissue destruction [16,17,18], baseline concentrations correlated with the degree of trauma and even exceeded those in patients with traumatic spinal cord injury [16], which is indicative of the excessive damage and systemic stress response to burn injury [34,35]. As shown in the subgroup analysis, initial SELENBP1 levels were particularly elevated in patients with a TBSA ≥ 30% and burns reaching the subcutaneous tissue (full-thickness burns). Considering the predominantly cytoplasmic localization of SELENBP1 in mature adipocytes under normal conditions [5], and its trauma-associated release into the systemic circulation, this interrelation becomes apparent and supports the notion of assessing serum SELENBP1 levels as a biomarker of burn severity. The greater elevation of baseline SELENBP1 among female patients is most probably attributable to their significantly higher burn severity as compared with male patients. Apart from that, no clear sex-specific differences concerning the dynamics and predictive value of SELENBP1 were found. This concurs with a previous trial in 75 patients undergoing cardiac surgery, which also reported only marginal sex-specific differences with regard to SELENBP1 kinetics [18].

After traumatic injury, the innate immune system induces a profound pro-inflammatory response, potentially aimed at promoting wound healing and mitigating the risk of microbe invasion and secondary infections. However, recurrent activation of the inflammatory cascade related to clinical procedures and complications can trigger a harmful “two-hit” response, which is common after major trauma and may predispose the host to opportunistic infections [36,37,38]. Acute burn injury leads to a similar release of pro-inflammatory cytokines, with peak levels persisting throughout the first week after the burn [39,40]. Likewise, inflammatory parameters, such as WBC counts, have been measured as being initially elevated in the serum of burn patients [41]. Consistent with these reports, we found serum SELENBP1 levels to positively correlate with inflammatory markers, i.e., WBCs and PCT, at admission. Baseline SELENBP1 concentration was not significantly associated with CRP, most likely due to the delayed onset of the acute phase response [42]. Unlike the previous research on circulating levels of SELENBP1, the present trial provided a prolonged follow-up period, allowing for an assessment of SELENBP1 dynamics up to six months after injury. We observed a rapid decrease in serum concentrations within the first day postburn. Interestingly, serum levels subsequently remained constantly low and did not show a “second hit”, despite extensive surgical procedures and the development of secondary infections. The underlying mechanism may reflect the reciprocal functional interaction between SELENBP1 and GPx-1, as previously described [12]. GPx-1 is a most abundant and ubiquitously expressed Sec-containing selenoenzyme that reduces cellular oxidative stress by limiting hydrogen peroxide accumulation [43,44]. GPx-1 ranks low within the hierarchy of selenoproteins and thus responds sensitively to Se supply or Se decline [45]. Therefore, high-dose intravenous Se supplementation in severely Se-depleted burn patients may have supported intracellular GPx-1 expression and prevented a second increase in serum SELENBP1. However, tissue samples were not accessible for testing this hypothesis in the current study.

Aside from local inflammation, hypoxia is a common finding in the burn wound microenvironment. While short-term hypoxia contributes to the initiation of wound healing, chronic hypoxia exerts numerous detrimental effects at both a cellular and a systemic level. Hypoxia leads to the formation of reactive oxygen species (ROS), which, in turn, mediate the activation of the transcription factor HIF-1 [46,47]. The expression of GPx-3, a major extracellular ROS scavenger, is transcriptionally upregulated by hypoxia through a HIF-1-binding site [48]. Notably, SELENBP1 also seems to interact with HIF-1; the mouse homolog of SELENBP1 has been identified as a HIF-1 target gene [49], and SELENBP1 has been shown to negatively regulate the alpha subunit of HIF-1 in LNCaP prostate cancer cells [50]. Despite the immediately impaired Se status following burn injury, correlation analysis of serum SELENBP1 did not reveal significant inverse associations with GPx-3 during the first days postburn, as opposed to Se and SELENOP. Since serum SELENBP1 levels and oxidative stress are both directly related to burn severity, the less pronounced decrease of GPx-3 in patients with higher SELENBP1 levels may result from hypoxia-induced GPx-3 expression. This finding appears to coincide with the results of a previous study where patients with septic shock exhibited a greater decline in serum SELENOP as compared with GPx-3 [51]. An analysis of SELENBP1 expression levels in relation to the local inflammatory response and the extent of hypoxia in the burn wound could provide useful information on the functional role of SELENBP1 in this context.

In line with our hypothesis, high concentrations of serum SELENBP1 at admission were predictive of pulmonary infection risk in burn patients, albeit not as accurately as overall burn severity, i.e., the ABSI. Infectious complications following major burns are largely driven by the enormous production of ROS and an impaired antioxidant defense [52]. GPx-1 deficiency in transgenic mice led to an increased susceptibility to ROS-induced cellular damage [53]. Accordingly, knockdown of SELENBP1 in HeLa cervical cancer cells decreased ROS levels and enhanced GPx-1 expression [54]. Based on these findings, one may speculate on an indirect aggravation of oxidative stress through intracellular SELENBP1 and that the shift of SELENBP1 to the intravascular space serves as a protective mechanism against oxidative damage and related post-acute sequelae. Still, this hypothesis and the physiological role and nature of the postulated SELENBP1-GPx-1 interaction in critically ill patients has yet to be elucidated.

Overall, this is the first study to assess the dynamics of serum SELENBP1 in severely burned patients and its linear correlation with the injury severity. Our study has certain strengths, including its relatively large and well-characterized patient cohort, the long-term follow-up, and the sensitive and robust immunoassay for SELENBP1 quantification. However, primarily as a consequence of its observational epidemiologic design, this study is not suitable for inferring mechanistic insights, such as the proposed SELENBP1-GPx-1 interaction, for which further molecular analyses are needed. Serum SELENBP1 kinetics in burn patients unexposed to supplemental Se and elevated GPx-1 activities cannot be deduced from this study, as supplemental Se was part of the routine clinical care of burn patients. The statistical power of this research is limited by the increasing number of missing values throughout the follow-up and the non-inclusion of additional, potentially confounding, variables in the multivariate analysis that are predictive of pulmonary infection risk in burn patients, e.g., pre-existing nutritional and immune status, comorbidities, timing of burn wound excision, and further supportive therapeutic measures. Furthermore, the analysis of sex-specific differences was limited by the small percentage of female patients.

Even though the association of serum SELENBP1 levels at admission with pulmonary infection risk did not reach statistical significance in our study, its immediate and strong elevation as well as its stringent positive correlation with burn severity indicate the potential relevance of this poorly characterized parameter in major trauma. Multicenter clinical trials and in vitro studies are warranted to allow greater insight into the regulation and function of SELENBP1 under conditions of hypoxia and inflammation. With a better understanding of the underlying cellular and molecular mechanisms, serum SELENBP1 may become a promising biomarker for estimating trauma severity and for identifying burn patients already at high risk of adverse clinical outcome at the time of hospital admission, who might benefit from intensified infection prevention measures.

## 5. Conclusions

Serum SELENBP1 concentration reflects the severity of trauma in burn patients and shows considerable associations with the risk of pulmonary infection postburn. Hereby, it complements the established parameters of injury like the ABSI and may contribute to a refined early assessment of damage severity and infection risk in severely burned patients. Further studies are required to elucidate the interplay between redox homeostasis, inflammatory response, serum and intracellular SELENBP1 changes, and clinical outcome in burn injury in more detail, and to provide a better overview of the value of the different biomarkers.

## Figures and Tables

**Table 1:** Baseline characteristics and clinical outcomes.

Variables	Pulmonary Infection (*n* = 37)	Other Infections (*n* = 22)	No Infection (*n* = 31)	Total (*n* = 90)
Sex Female Male	8 (21.6) 29 (78.4)	6 (27.3) 16 (72.7)	3 (9.7) 28 (90.3)	17 (18.9) 73 (81.1)
Age (yr)	51.4 ± 17.9	40.6 ± 17.4	42.4 ± 16.1	45.7 ± 17.7
BMI (kg/m^2^)	24.9 (22.5–28.4)	27.2 (22.7–29.9)	26.2 (22.8–30.8)	25.7 (22.6–29.3)
TBSA (%)	35.0 (26.0–40.0)	30.0 (22.9–50.8)	20.0 (13.5–29.5)	29.0 (20.0–37.0)
Full-thickness burn	26 (70.3)	16 (72.7)	10 (32.3)	52 (57.8)
Inhalation injury	15 (40.5)	4 (18.2)	3 (9.7)	22 (24.4)
ABSI	8.0 (7.0–10.0)	7.0 (6.0–9.0)	6.0 (4.0–7.0)	7.0 (6.0–9.0)
SELENBP1 (µg/L)	45.6 (21.2–70.5)	35.8 (8.7–61.6)	12.5 (3.7–34.2)	26.9 (10.0–61.0)
Sepsis	36 (97.3)	19 (86.4)	0 (0.0)	55 (61.1)
Mortality	3 (8.1)	1 (4.5)	1 (3.2)	5 (5.6)
Length of stay (d)	54.0 (21.0–87.0)	31.5 (23.3–44.8)	20.0 (10.0–25.0)	27.0 (19.0–56.0)
Length of ICU stay (d)	32.0 (17.0–54.0)	18.5 (13.3–27.0)	8.0 (2.0–14.0)	17.5 (8.3–40.8)

TBSA, total body surface area; ABSI, Abbreviated Burn Severity Index.

**Table 2:** SELENBP1 correlations over time.

SELENBP1 Correlations (*p* < 0.05)							
	D0	D1	D2	D3	W1	W2	Full
Se		–0.282	–0.227				–0.230
SELENOP	–0.382	–0.305	–0.303				–0.210
GPx-3							–0.170
Cu							–0.127
Zn							–0.124
WBCs	0.421	0.249		–0.240			0.197
CRP			0.260				–0.243
PCT	0.340	0.253	0.391		0.228		

Se, selenium; SELENOP, selenoprotein P; GPx-3, glutathione peroxidase 3; Cu, copper; Zn, zinc; WBCs, white blood cells; CRP, C-reactive protein; PCT, procalcitonin. Significant Spearman correlation coefficients are presented (green: positive correlations; orange: negative correlations).

**Table 3:** Validity of baseline SELENBP1 and the ABSI for the prediction of pulmonary infection.

	Cut-Off Point	Sensitivity (95% CI)	Specificity (95% CI)	PPV	NPV
SELENBP1	>21.2 µg/L	75.7 (59.9–86.6)	64.5 (47.0–78.9)	59.8	79.2
ABSI	>6.5	86.5 (72.0–94.1)	71.0 (53.4–83.9)	67.5	88.3

ABSI, Abbreviated Burn Severity Index; CI, confidence interval; PPV, positive predictive value; NPV, negative predictive value.

**Table 4:** Multiple logistic regression.

	OR	95% CI	*p*-Value
Baseline SELENBP1 > 21.2 µg/L	2.47	0.68–8.88	0.164
ABSI > 6.5	14.37	3.15–88.78	0.001
Age	0.99	0.94–1.03	0.574
Sex (Female)	1.41	0.26–9.50	0.707
Intercept	0.25	0.03–1.66	0.161

ABSI, Abbreviated Burn Severity Index; OR, odds ratio; CI, confidence interval.

## Data Availability

The data presented in this study are available on request from the corresponding author. The data are not publicly available due to containing patient confidential information.
